# Gemini Surfactants Based on Bis-Imidazolium Alkoxy Derivatives as Effective Agents for Delivery of Nucleic Acids: A Structural and Spectroscopic Study

**DOI:** 10.1371/journal.pone.0144373

**Published:** 2015-12-07

**Authors:** Zuzanna Pietralik, Żaneta Kołodziejska, Marek Weiss, Maciej Kozak

**Affiliations:** 1 Department of Macromolecular Physics, Faculty of Physics, Adam Mickiewicz University, Umultowska 85, 61–614 Poznań, Poland; 2 Institute of Physics, Poznań University of Technology, Piotrowo 3, 60–965 Poznań, Poland; 3 Joint Laboratory for SAXS studies, Faculty of Physics, Adam Mickiewicz University, Umultowska 85, 61–614 Poznań, Poland; University of Quebec at Trois-Rivieres, CANADA

## Abstract

The success rate of gene therapy depends on the efficient transfection of genetic material into cells. The golden mean between harmlessness and high effectiveness can be provided by synthetic lipid-like molecules that are similar to the components of biological membranes. Cationic gemini surfactants are one such moiety and because of their favourable physicochemical properties (double positive electric charge, reduced toxicity, low values of critical micelle concentration), they show great potential as delivery system components for genetic material in gene therapy. The aim of this study was to investigate the process of the complexation of cationic gemini surfactants with nucleic acids: double-stranded DNA of different sizes (21 bp, ~185 bp, ~20 kbp) and siRNA (21 bp). The tested series of dicationic surfactants consists of bis-imidazolium quaternary salts with varying lengths of hydrophobic side chains (*m* = 5, 6, 7, 8, 9, 11, 12, 14, 16). On the basis of the data obtained by circular dichroism spectroscopy and electrophoresis, we concluded that the studied gemini surfactants with long side chains effectively bind nucleic acids at low concentrations, which leads to the formation of stable lipoplexes. Images obtained by atomic force microscopy also confirmed the formation of vesicular structures, i.e., complexes between DNA and surfactants. The cytotoxicity of selected surfactants was also tested on HeLa cells. The surfactant toxicity significantly depends on surfactant geometry (the length of hydrophobic chain).

## Introduction

Modern therapeutic approaches should not focus solely on treating the symptoms and mitigating the effects but also extend to the molecular origins of disease. Knowledge of the aetiology of a particular disease can enable the correction of organism’s dysfunction, stop the disease’s progression and (sometimes) even completely undo its effects without the side effects associated with standard (not personalized) pharmacological therapy [1,2]. Very promising therapeutic pathways that permit the repair of an organism through the adjustment or replacement of genetic information are referred to as gene therapy [3,4]. introducing therapeutic copies of genes (DNA or RNA fragments) into the organism; the genes then condition the cells’ correct functioning Gene therapy has proven successful not only in treating severe illnesses such as Leber’s congenital amaurosis [5] and Parkinson’s disease [6] but also in providing antitumor treatment [7]. Essentially, gene therapy is a set of therapeutic methods based on replacing a defective gene with a healthy one, completing a missing gene to express the required protein [3] or constraining the expression of a particular gene (using the mechanism of RNA interference, RNAi) [8,9].

Transfection, i.e., the process of delivering genetic material into cells, is the underlying mechanism of gene therapy. The most straightforward way to deliver genetic material can be achieved by direct transfer of "naked" DNA via injection [10]. However, this procedure is inferior to other means of application because it is both inefficient and nonselective. Therefore, for the purpose of prevalence, it has become necessary to use delivery systems called vectors, which protect and ensure delivery of genetic material to the targeted cells. Viral-based vectors show extremely high transfection efficiency [4, 11], but because of those vectors’ recognized side effects (e.g., oncogenesis) and undesirable immune response, researchers must search for new solutions. As an alternative to viruses, cationic molecules such as polymers (polyplexes) or amphiphilic compounds (lipoplexes) can be used [10,12]. Each of these vectors possesses numerous unique features, and each vector has its own advantages and disadvantages. The elementary criterion for selecting the appropriate non-viral vector is the capability to bind the nucleic acid (DNA or RNA) and to release genetic material to a particular destination and under specific conditions. Naturally, a vector must fulfil numerous requirements, such as biocompatibility, biodegradability, and well-defined chemical and physical properties.

Cationic gemini surfactants (also known as dicationic, dimeric or Siamese surfactants) are often reported to be a promising alternative to virus-based vectors; they are being widely tested for use in gene therapy [13–17]. This group of surfactants consists of amphiphilic molecules, which can be described as a dimer of two monomeric surfactant moieties because they are composed of two aliphatic side chains (hydrophobic part) and two cationic heads (hydrophilic part) separated by a rigid or flexible linker (called a spacer). These surfactants typically show greatly enhanced properties in comparison to their corresponding monovalent (single chain, single head group) compounds [13,14]. The advantages of these amphiphiles include significantly increased surface activity and CMC values (critical micelle concentration) reduced by the order of magnitude. They also tend to form a variety of aggregates in solutions, which is beneficial for many possible applications. Most of all, because of their unique structure (i.e., their amphiphilic character and their two positive charges located in the single molecule), gemini surfactants interact effectively with DNA on the electrostatic bases at very low concentrations, leading to the entrapment and condensation of nucleic acid. It has been shown that the dicationic surfactant can effectively bind DNA, thus forming complexes (lipoplexes) in which nucleic acid is protected and can be transported [15–17]. Moreover, they exhibit very low toxicity compared to many monomeric surfactants, transfection efficiencies as high as 90% [18] and the ability to transfect numerous cell lines that are normally difficult to transfect.

Specific built of gemini surfactants allows to achieve almost endless possibilities of compositions for these molecules. The most extensively studied group of gemini surfactants is a series of N,N-bis(dimethylalkyl)-α,ω-alkanediammonium dibromides, [19,20], for which *m*-*s*-*m* denotation is used (in which *m* represents the length of the hydrophobic side chain, and *s* is the number of segments in the polymethylene spacer group). The length of spacer group (*s*) greatly modifies the physicochemical properties of gemini surfactants [13,14]. Consequently, the most common aspect investigated in gemini surfactant-based vectors is the influence of spacer length on the ability to bind nucleic acids [18, 21, 22]. The aspect of side chains length is though scattered throughout different studies, focusing mostly on amphiphiles containing between 12 and 18 carbons in chains. For example, a series of surfactants denoted as 12-*s*-12 with spacer length *s* = 3, 6 and 12 carbon atoms has been tested as a vehicle for transporting siRNA [22, 23]. A series of surfactants denoted as 12-*s*-12 with a wider range of linker lengths, i.e., *s* = 3, 4, 6, 8, 10, 12, 16 and 16-3-16, have been investigated as complexing agents for plasmid DNA [24]. Additionally, another group of surfactants denoted as 16-*s*-16 and 16:1-*s*-16:1 with *s* = 2, 3, 6 (where 16:1 signifies a 16-carbon chain with one double bond) have been reported as good reagents for mediating DNA transfection [25]. Lipoplexes consisting of different dicationic compounds, where *m* = 12, *s* = 3, 12 and *m* = 18:1 (oleyl); *s* = 2, 3, 6; and salmon sperm DNA have also been characterized [26]. In addition, cationic gemini surfactants containing polyoxyethylene spacers (with oxyethylene repeats of different lengths (C_2_H_4_O)_*n*_, where *n* = 1, 2 or 3) have been tested as improved transfecting agents of plasmid DNA in cancer cells [27].

The aim of our work was to investigate the influence of the hydrophobic alkoxymethyl side chain length of gemini surfactants on the ability to effectively bind nucleic acids and form stable complexes, which could potentially be used for future therapeutic tests. Therefore, we tested a series of dicationic surfactants with chains of different polymethylene lengths, with *m* ranging from 5 to 16. The series of dicationic surfactants consisted of bis-imidazolium quaternary salts because it has been reported that an amphiphile-containing imidazolium moiety exhibits low toxicity and is more likely to be applicable as a synthetic vector [21]. For example, a series of imidazolium-based surfactants, hexadecyl chains and spacer groups with *s* = 3, 4, 5, 6, 8 have been examined as vectors for plasmid DNA [21]. In gene therapy, a variety of forms of nucleic acids (circular or linear DNA as well as siRNA) have been used; their size and form depends on the particular disease-treatment procedure. Therefore, to investigate the process of complexation thoroughly, four different nucleic acids were tested: DNA varying in size (21 bp, ~185 bp, ~20 kbp) and siRNA (21 bp).

To evaluate the above-described synthetic vectors as delivery systems for gene therapy, experiments using agarose gel electrophoresis, cytotoxicity tests, circular dichroism (CD) spectroscopy and atomic force microscopy (AFM) were carried out. The insight that we obtained into the physico-chemical properties of obtained lipoplexes has proven to be essential to understanding the link between efficiency and low cytotoxicity while also elucidating the behaviour of lipoplexes in the biological environment.

## Materials and Methods

**Fig 1:**
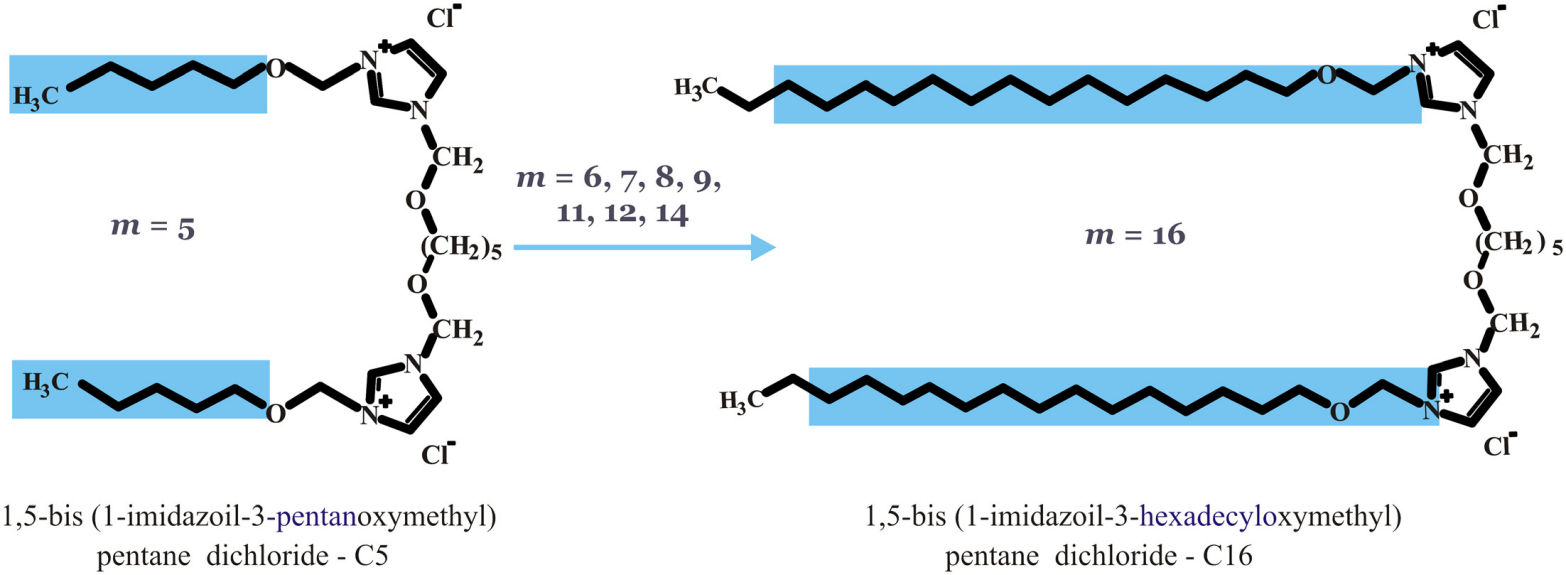
Chemical structures of studied gemini surfactants, i.e., alkoxyderivatives of bis-imidazolium quaternary salts with different-length hydrophobic side chains (*m* = 5, 6, 7, 8, 9, 11, 12, 14, 16).

A series of dicationic gemini surfactants 1,5-bis(1-imidazolilo-3-alkoxymethyl) pentane dichloride were synthesized according to a procedure reported previously [28] and were a generous gift from dr Andrzej Skrzypczak (Poznań University of Technology, Poland). Surfactants differ in the length of alkoxymethyl side chains, ranging from pentanoxymethyl to hexadecyloxymethyl, as indicated by their designations (C5, C6, C7, C8, C9, C11, C12, C14 and C16). The chemical structures of the gemini surfactants studied are presented in Fig 1.

The ability to form complexes was tested on four different nucleic acids. Two types of model nucleic acid from salmon sperm, specifically low molecular weight DNA (~185 bp) and high-molecular-weight DNA (~20 kbp), were purchased from Sigma-Aldrich. Small double-stranded DNA and RNA oligomers consisting of 21 base pairs were purchased from FUTURE Synthesis (Poland). All of the nucleic acids were used without further treatment.

### Lipoplex preparation

The lipoplexes were prepared by directly mixing gemini surfactant solutions with nucleic acid solutions in a volume ratio 1:1, keeping the concentration of the latter constant to obtain specific values for the *p/n* ratio, which is defined as the ratio of positive (gemini surfactants) to negative electric charge (nucleic acids). The studies were conducted for the following set of *p/n* values: 0.5, 0.75, 1, 2, 3, 4, 5, 6, 8, 10, 12, 14, 16, 18, 20, 25, 30, 35 and 40. These values correspond to the following molar concentrations of gemini surfactants: 0.2, 0.3, 0.4, 0.8, 1.2, 1.6, 2, 2.4, 3.2, 4, 4.8, 5.6, 6.4, 7.2, 8, 10, 12, 14, and 16 mM. Solutions of nucleic acids were prepared by dissolving powdered nucleic acids in 10 mM phosphate buffer (pH 6.8) to obtain starting concentrations of 38 μM of 21 bp nucleic acids, 4.3 μM of 185 bp DNA and 0.04 μM of 20 kbp DNA. Homogenous 32 mM solutions of all of the studied gemini surfactants were prepared by dissolving crystalline powders in dH_2_O; this was followed by 5 minutes of sonication at 50°C. Next, series of dilutions were prepared covering concentrations ranging from 32 mM to 0.4 mM. All of the samples were incubated for 15 minutes at room temperature prior to measurement.

### Electrophoresis

The binary surfactants/nucleic acids systems were subjected to agarose gel electrophoresis in the presence of ethidium bromide (0.5 μg/ml). To achieve good separation, 0.8% gels were prepared for the high-molecular-weight DNA, 1.5% gels for low-molecular-weight DNA and 2% gels for 21 base-pair DNA and RNA. All of the gels were prepared by dissolving appropriate amounts of agarose in Tris/Borate/EDTA (TBE) buffer (90 mM Tris base, 90 mM boric acid, 2 mM disodium EDTA, pH 8). Loading solution consisting of 0.25% bromophenol blue, 0.25% orange G and 40% glucose in a 1× TBE buffer was added at a volume of 4 μl per 20 μl sample. The 10 μl samples were placed in gel wells, including the reference samples, which contained pure nucleic acid solutions and a wide-range DNA marker (Sigma Aldrich). Electrophoresis was carried out at 120 V for 1 h or until the dye approached the bottom of the gel. The gels were illuminated using a standard UV transilluminator and 300 nm wavelength radiation; the electrophoretic mobility patterns obtained were recorded using a Syngene G:BOX imaging system.

### Circular dichroism spectroscopy

To determine whether structural changes in DNA or RNA occurred upon binding of cationic surfactants to nucleic acids, circular dichroism spectra were recorded on a J-815 JASCO spectropolarimeter. All CD measurements were carried out at room temperature (25 ± 1°C) using a 1 mm quartz cuvette. Scans were obtained in the UV range between 220 and 350 nm, at a scan rate of 100 nm/min with bandwidth of 1 nm and integration time of 2 s. Each time, five subsequent spectra were averaged to improve the signal-to-noise ratio. The background CD spectrum recorded for the buffer solution was subtracted from the samples spectra, which were then smoothed using the Savitzky-Golay function over 9 points. All of the data were analysed using Spectra Manager II (Jasco) and Origin software.

### Atomic force microscopy

Atomic force microscopy (AFM) has been used to observe the topography and shape of complexes composed of nucleic acids and surfactants. The sample solutions (5 μl) were adsorbed on a freshly cleaved mica surface, rinsed with dH_2_O and dried at room temperature (25 ± 1°C) before visualization. Samples were examined in intermittent air contact mode by diInnova (Veeco) AFM with a large-area, closed-loop scanner (100×100 μm). For this purpose, PPP-NCLR (Nanosensors) silicon micro-cantilevers with tip radius below 7 nm were used. The recorded AFM images were analysed using Gwyddion software (version 2.31) [29].

### MTT assay

Cytotoxicity of the studied gemini surfactants at various concentrations was determined using the standard MTT Cell Proliferation Assay Kit (Cayman Chemicals, Michigan, USA), based on protocol described by Mossmann [30], and HeLa cell line. The cells were cultivated using a standard protocol [31], at 37°C, 85% relative humidity, and 5% CO_2_, in DMEM supplemented with 10% FCS. Cells were seeded into 96-well plates and they were incubated with the appropriate solutions of surfactant diluted with standard cell culture medium in proportion 1:100, for 1 h and then visualised with Zeiss Axiovert microscope (Carl Zeiss, Oberkochen, Germany). After applying MTT assay protocol, absorbance at 570 nm was measured using Tecan Infinite 200 pro microplate reader (Männedorf, Switzerland).

## Results and Discussion

**Fig 2:**
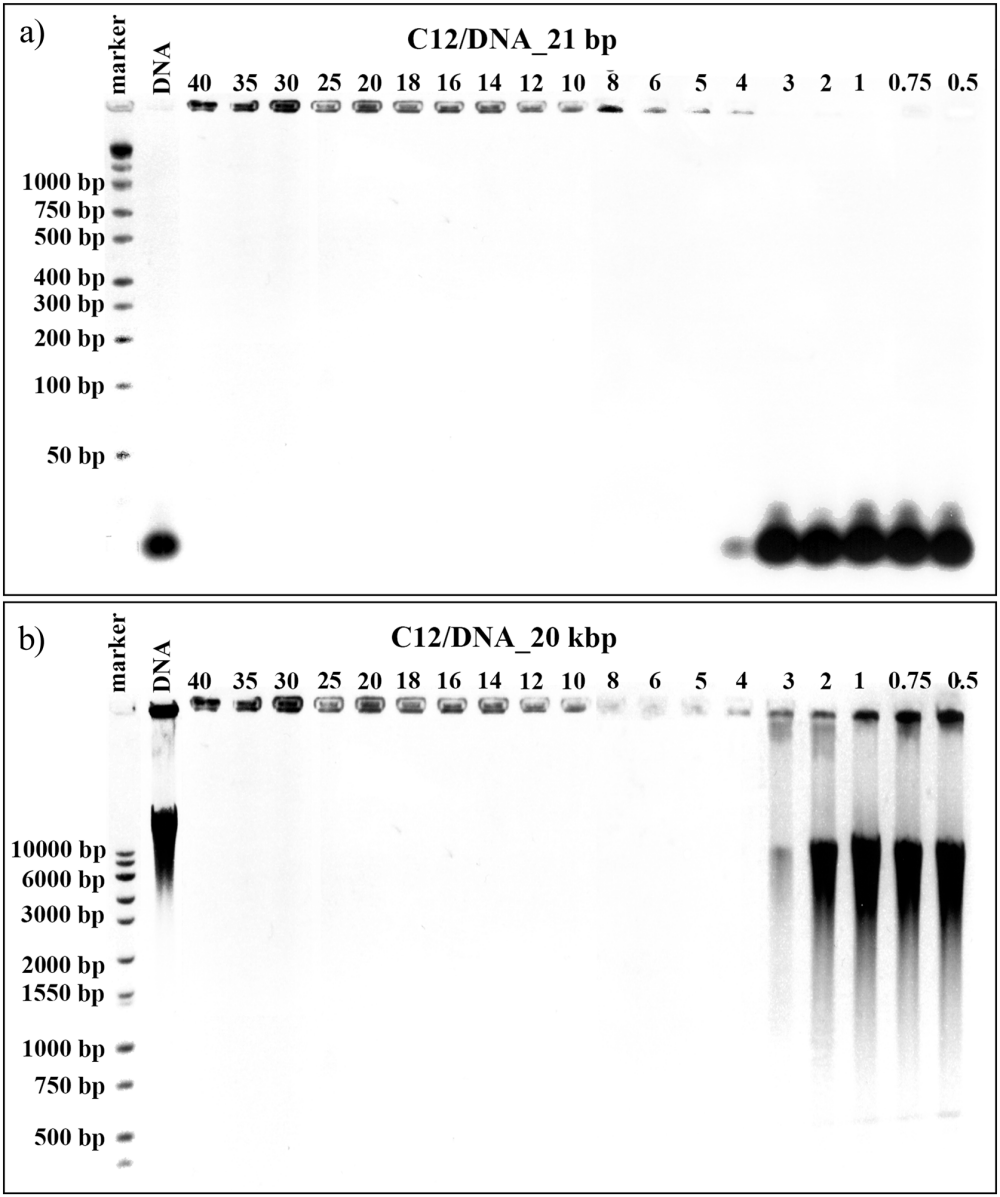
Agarose gel electrophoresis results. Images obtained for lipoplexes based on C12 gemini surfactant and duplex DNA consisting of 21 bp (a) and for lipoplexes based on C12 gemini surfactant and high-molecular-weight salmon-sperm DNA consisting of 20 kbp.

The first technique, which was used to characterise the process of lipoplex formation, was agarose gel electrophoresis in the presence of ethidium bromide. This technique is fairly basic and elementary, yet it provides valuable information about tested systems (e.g. on the success of lipoplex formation). Therefore, it was applied in our studies as a preliminary trial for judging both which surfactants and at which values of *p/n* charge ratios form complexes with particular forms of nucleic acids. Once a stable complex of surfactant and nucleic acid is formed, the complex does not migrate under applied voltage. For tested lipoplexes, the *p/n* charge ratio range was limited to values from 0.5 to 40 because of the possible cytotoxicity of the studied surfactants at higher concentrations. This corresponds to the maximum tested concentration of a surfactant studied equal to 16 mM. The results of the electrophoresis experiments performed on lipoplexes formed in systems consisting of surfactant C12 with either one small 21 bp DNA or one high-molecular-weight DNA (20 kbp) are presented in Fig 2.

The standard sample—DNA molecular weight marker is visible in the first lane in both images, enabling a determination of the mass of nucleic acid located in the subsequent lanes. The pure nucleic acid solutions were also used as reference samples, and based on those samples, the natural electrophoretic mobility of DNA and RNA was evaluated. For binary systems prepared on the basis of 21 base pair DNA oligomers (Fig 2a), we observed no formation of complexes with charge ratios between 0.5 and 3 in which the effective interactions between the surfactant and the nucleic acid were insufficient. In the case of the sample characterized by *p/n* = 4, the majority of the DNA oligomer was unable to migrate through the gel and remained in the well, indicating that the DNA was located in a complex with the surfactant, although some traces of free DNA molecules were still visible. For higher surfactant concentration, i.e., values of *p/n* > 5, stable complexes were formed and the entire DNA package was bounded. Complete complex formation is extremely important because, like most biological molecules, DNA in its free form is degraded rapidly in the body, which may cause an immune response [32,33]. Its encapsulation into a delivery system provides protection from nucleases, therefore increasing circulation time and permitting accumulation at the desired destination. The same surfactant (C12) appeared to be more efficient in the case of the largest DNA tested (20 kbp–Fig 2a) because partial complexation was visible in the sample with *p/n* = 3 and full complexation was visible in the sample with *p/n* = 4.

Analogously, the investigation was conducted throughout the range of concentrations for all tested dicationic surfactants with four different nucleic acids. The results of electrophoretic tests for all of the studied lipoplexes are summarized in Table 1. demonstrating the relationship between the charge ratios *p/n* and the formation of stable complexes. For samples in which only partial complexation occurred (such as in the sample C12/DNA_21 bp, *p/n* = 4), the point was not taken into account.

**Table 1:** Summary of the results of electrophoretic experiments on the studied series of gemini surfactants and all nucleic acids tested, demonstrating the relationship between the charge ratio *p/n* characterizing the systems and the formation of stable complexes. Denotation 20 kbp indicates the complete complex formation in the case of 20 kbp salmon-sperm DNA, denotation ***185 bp*** shows under which conditions complexes were formed with low-molecular-weight salmon-sperm DNA (185 bp), and denotation **21 bp** designates the complexes in systems with the smallest tested nucleic acids (both 21 bp DNA and RNA).

*p/n| surf*	C5	C6	C7	C8	C9	C11	C12	C14	C16
0.5	-	-	-	-	-	-	-	-	-
0.75	-	-	-	-	-	-	-	-	-
1	-	-	-	-	-	-	-	-	-
2	-	-	-	-	-	-	-	-	-
3	-	-	-	-	-	-	-	-	-
4	-	-	-	-	-	-	20 kbp	-	-
5	-	-	-	-	-	***185 bp***	**21 bp**	20 kbp	20 kbp
6	-	-	-	-	-	***185 bp***	**21 bp**	20 kbp	20 kbp
8	-	-	-	-	-	**21 bp**	**21 bp**	***185 bp***	***185 bp***
10	-	-	-	-	-	**21 bp**	**21 bp**	***185 bp***	**21 bp**
12	-	-	-	-	-	**21 bp**	**21 bp**	**21 bp**	**21 bp**
14	-	-	-	-	20 kbp	**21 bp**	**21 bp**	**21 bp**	**21 bp**
16	-	-	-	-	20 kbp	**21 bp**	**21 bp**	**21 bp**	**21 bp**
18	-	-	-	-	20 kbp	**21 bp**	**21 bp**	**21 bp**	**21 bp**
20	-	-	-	-	20 kbp	**21 bp**	**21 bp**	**21 bp**	**21 bp**
25	-	-	-	-	***185 bp***	**21 bp**	**21 bp**	**21 bp**	**21 bp**
30	-	-	-	-	**21 bp**	**21 bp**	**21 bp**	**21 bp**	**21 bp**
35	-	-	-	-	**21 bp**	**21 bp**	**21 bp**	**21 bp**	**21 bp**
40	-	-	-	-	**21 bp**	**21 bp**	**21 bp**	**21 bp**	**21 bp**

**Fig 3:**
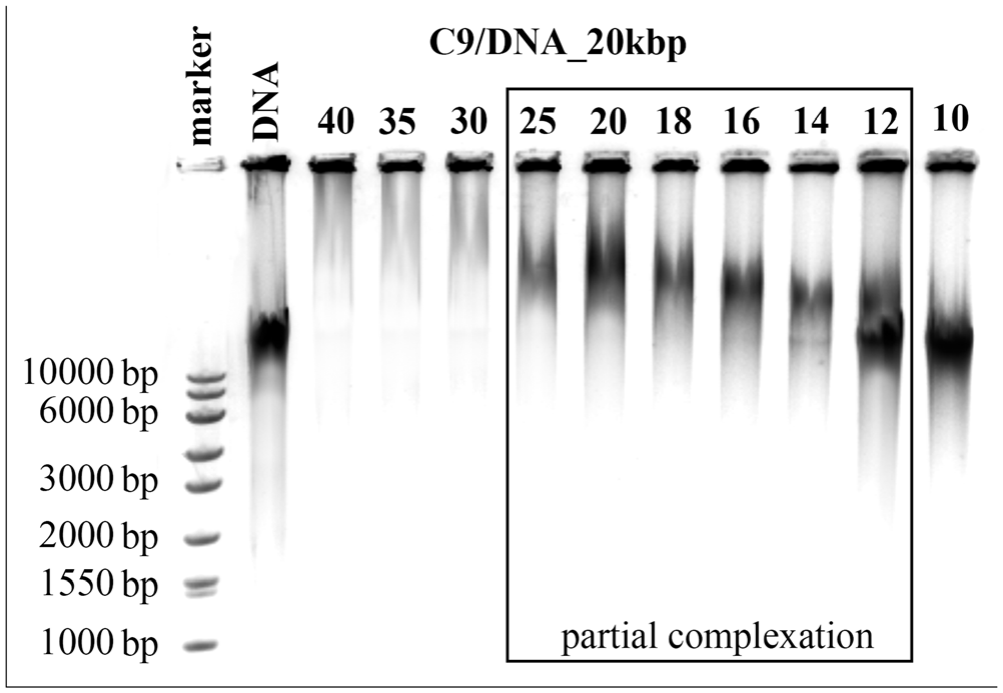
Agarose gel electrophoresis of lipoplexes based on C9 surfactant with high-molecular-weight salmon-sperm DNA (20 kbp).

Based on the obtained results, we concluded that surfactants with short side chains, i.e., those containing fewer than 9 carbon atoms (nonyloxymethyl side chain group) in the side chains, were completely ineffective at binding any nucleic acids in the tested range of concentration. Increasing the *p/n* ratio could possibly lead to complex formation, but it would also increase the toxicity of the lipoplexes, which contradicts our goals. The first signs of complex formation were visible for surfactant C9, for which the complexes with 20 kbp DNA were formed at *p/n* = 14, with 185 bp DNA at *p/n* = 25 and 21 bp nucleic acids at *p/n* = 30. In the case of this surfactant, the biggest difference in the complexation of various nucleic acids was observed. One explanation for this result may be that C9 surfactant leads to the partial complexation of nucleic acid in a wide range of *p/n* values, a result that is visible in Fig 3.

An increased concentration of the surfactant in the system have caused an increase in the size of the nucleic acid, which would mean that some surfactant molecules interact with DNA and disable nucleic acid molecules from moving with their natural electrophoretic mobility. This phenomenon can be seen in the *p/n* range 12–25; therefore, the boundary conditions were assessed for 100% of free DNA molecules in the system with *p/n* = 10, whereas those conditions were assessed for 100% of the DNA in the complex with *p/n* = 30. This behaviour was characteristic for only the C9 surfactant; for example, for small oligomers, the estimated size changes were in the range of 30–1500 bp, based on the smearing electrophoretic pattern obtained.

The longer the side chains, the more efficient surfactants were at forming complexes and the differences among the binding processes of nucleic acids of different sizes was narrower. The addition of two methyl groups to the side chains significantly improved the surfactant’s binding ability; thus, the C11 surfactant had bound high-molecular-weight DNA at charge ratio *p/n* = 5, a low MW DNA at *p/n* = 8 and oligonucleotides (21bp siRNA and dsDNA) at *p/n* = 8. The result is interesting: the length of the side chains of the surfactants reached its optimum at 12 carbon atoms in alkyl chains (dodecyloxymethyl side chain group) because C12 surfactant was the most efficient at complexing all of the tested nucleic acids. The *p/n* values required for 100% complex formation were 4 for both low and high MW salmon-sperm DNA and 5 for both of the oligomers tested. For longer side chains, the required quantity of surfactant to bind nucleic acids needed to be higher, especially in the case of high MW salmon-sperm DNA (*p/n* = 12 for C14, *p/n* = 10 for C16).

In the literature, the most-studied systems based on gemini surfactants consist of either 16 or 12 methylene groups in the side chains [27,34]; the most explored aspects of these systems are the influence of the length of the spacer group [35,36] and recently, the character of both spacer [37] and side chains [38]. A few papers have noted the strong dependence of side-chain length on CMC values [34], but a thorough investigation of the correlation between side-chain length and the possible application of those chains as vectors for gene therapy has not been published. Nevertheless, it is well known that for high effectiveness, a spacer group of optimum length is required [18,21]. Therefore, the absence of a simple, linear dependence on side-chain length is unsurprising.

Another conclusion, which has arisen from electrophoretic experiments, is that the size of the nucleic acid used also affects the process of complexation. Thus, for short nucleic acids, complexation occurs for higher values of *p/n* ratios and the longer nucleotide sequences, the lower the values of *p/n* for which stable complexes were formed. For example, C14 surfactant was effective at a charge ratio of 12 for both 21 base-pair DNA and RNA oligomers, for larger DNA at a *p/n* of 8 and for the high-molecular-weight DNA (20 kbp) at *p/n* = 5. This can be explained by the condensation of larger DNA, which results in the DNA’s effective charge being much lower than DNA’s actual overall charge [38,39]. For smaller molecules, the effective charge that can interact with negatively charged surfactant molecules is probably more or less equal to the overall charge of the entire DNA molecule. Additionally, the smallest tested nucleic acids, i.e., RNA and DNA oligomers, have been proven to be equally effective, although the destination and the mechanism to achieve the therapeutic effect is different in their case. This may indicate that only the negative charges play a role in the process of complexation regardless of the type of nucleic acid and the specific base-pair sequence. Generally, other authors have also noted that lipoplex structure not only is determined by the properties of amphiphilic molecules but also is less dependent on nucleic-acid characteristics [40]. Many studies have discussed that the distance between the positive charges (located at the nitrogen atoms of the imidazolium rings) is crucial to the interactions with the negative charge of the nucleic acids’ phosphate group for forming the complexes [41]. In the case of studied series of dicationic surfactant, the spacer group should not be in the U-shape pointed toward the hydrophobic interior because it has been shown that the minimum length is more than 12 carbon atoms for that configuration [24]. Accordingly, we can assume that the distance between positive charges in all of the studied surfactant molecules is the same and therefore, the observed effect is attributed only to the length of the side chains. In the literature, the objects of study have been primarily surfactants with various lengths or types of spacer groups [27,33,36], all of which contain long aliphatic side chains that primarily consist of 12 [24,42], 14 [43] or 16 [24,36] carbon atoms in chains. Nevertheless, a thorough investigation of the influence of side-chain length is still needed. Only partial information has been provided in previous studies: for example, higher transfection efficiency was proven in cases of 16-3-16 than in cases of an entire series of 12-*n*-12 surfactants, but the authors connected it with the higher CMC value of the latter [24]. In addition, Zhou et al. [44] have noted that hydrophobic interaction plays an important role in the process of DNA condensation in the case of other bis-imidazolium surfactants (*n* = 10, 12, 14), and have demonstrated that the longer the tails, the less surfactant needed to form the complex.

After preliminary analysis, four of our surfactants (C11, C12, C14 and C16) were selected for further examination using circular dichroism spectroscopy. This technique is used to determine the conformational changes in DNA caused by the interactions with the studied gemini surfactants. The observed CD signal derives solely from the DNA base arrangements because the surfactant molecules do not make any contribution. The studied *p/n* range was further limited to values from 0.5 to 20, to reduce the cytotoxicity of formed lipoplexes in future applications for transfection. Therefore, the maximum tested concentration of surfactant was 8 mM. The values of the lowest surfactant concentrations and corresponding *p/n* ratios, both of which cause stable and complete complex formation, are summarized in Table 2.

**Table 2:** Summary of the lowest *p/n* values and concentrations of surfactant for which 100% complexes are created.

	RNA 21bp		DNA 21bp		DNA 185bp		DNA 20kbp	
Surfactant	*p/n*	M [mM]	*p/n*	M [mM]	*p/n*	M [mM]	*p/n*	M [mM]
C11	8	3.2	8	3.2	5	2	4	1.6
C12	5	2	5	2	4	1.6	4	1.6
C14	12	4.8	12	4.8	8	3.2	5	2
C16	10	4	10	4	8	3.2	5	2

**Fig 4:**
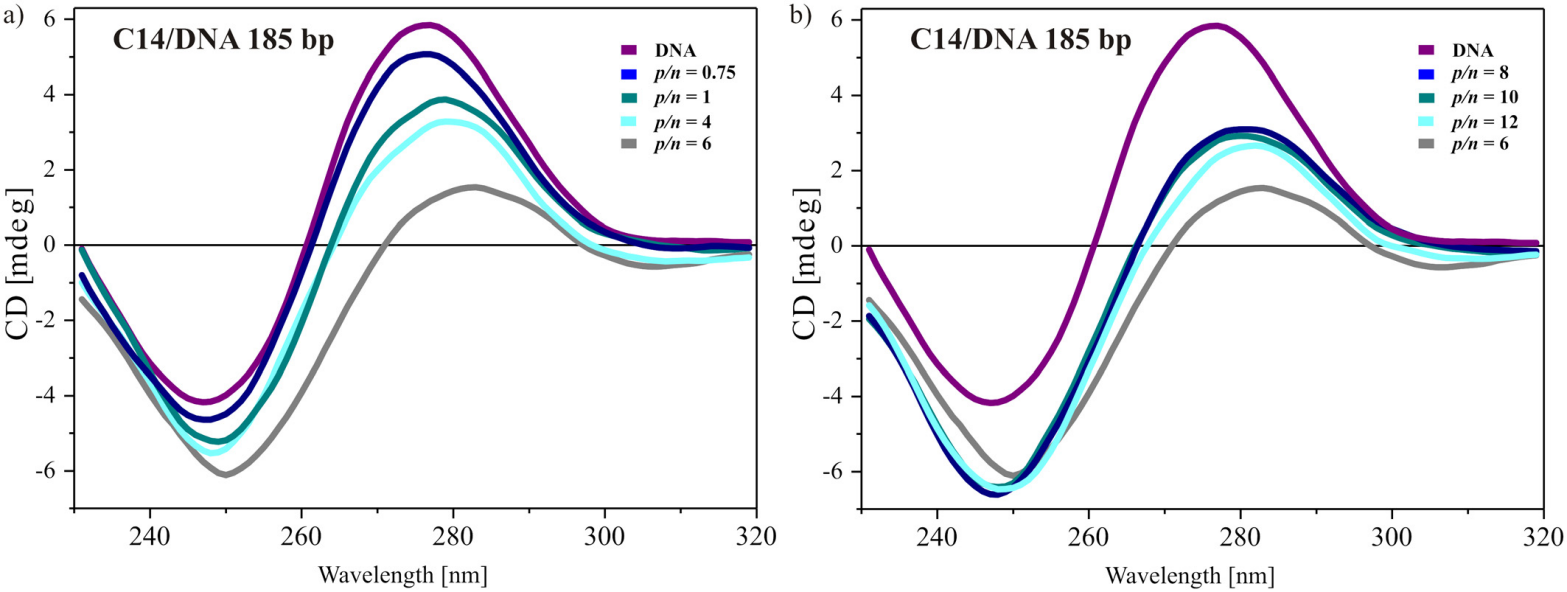
CD spectra of lipoplexes based on C14 and low-molecular-weight DNA under conditions ensuring complexation (b) and for lower *p/n* ratios (a).

In Fig 4, an exemplary CD spectral behaviour for binary studied systems of low-molecular-weight salmon-sperm DNA (185 bp) and surfactant C14 is presented. Fig 4a shows both the spectra that were recorded before reaching the full level of complexation and the spectrum obtained for the sample characterized by *p/n* = 6, which, based on the electrophoretic experiments, was the first sample containing 100% complexes. The spectrum recorded for the reference system, which was a 2 μM solution of DNA without the addition of the surfactant (violet curve), was characterized by a maximum at 278 nm and a minimum at 245 nm, with an intersecting point at 260 nm. These spectral features are indicative of the DNA molecules that form the typical native B-form in solution [26,45–47]. Even a small addition of studied gemini surfactant to the system was reflected in the CD spectral features. Based on Fig 4a, upon approaching the boundary conditions, the negative band increased simultaneously with a gradual decrease in the intensity of the maximum peak, and the spectra shifted towards a higher wavelength. For values of charge ratio 0.5 and 0.75, only the increase of the negative band and the decrease of the positive band were observed. For samples with higher surfactant content, both redshift and changes in intensity were detected. For *p/n* = 1, the shift of the spectrum was on the order of 2 nm, whereas for higher charge ratios *p/n* from 1 to 6, it reached 6 nm.

Additionally, the changes in the intensity of bands were progressive and for *p/n* = 6, the decrease in the intensity of the negative band reached 80% (20% of the initial value of the reference DNA solution) and the increase in the intensity of the positive band rose by 60%. The collapse of the positive band used to be wrongly attributed to the transition to the C-form of DNA, but more likely it is connected with forming the B-form with 10.2 base pairs per turn instead of the usual 10.4 [48]. The C-form would require a decrease in the negative band at 245 nm, which was not observed in our measurements. The other possibility is the transition of DNA into the highly condensed *ψ*-form [35,48], which is characterized by the following CD features: enhanced negative ellipticity, an overall shift of the bands towards higher wavelengths, flattening of the positive band and the appearance of long “tails” above 300 nm. The observed changes were not very pronounced and thus, they have been attributed to the condensation of nucleic acid and the changes in the hydration shell of the phosphate groups of DNA [44] following addition of surfactants to the system. It is likely that the monomer–monomer repulsion of DNA is also diminished by the cationic surfactants [16].

Fig 4b presents the CD spectra recorded for samples in which 100% of the nucleic acid are in a complex based on gel electrophoresis. This plot also contains both the reference DNA spectrum and the spectrum recorded for the system at *p/n* = 6, which was the boundary ratio for complex formation. For the systems with higher surfactant content, the redshift was in the order of 3 nm; the negative ellipticity was still increased by 60% but the positive ellipticity was also increased, reaching 50% of its initial value (obtained for the reference-DNA solution). CD spectra were unaltered even with the further addition of amphiphilic compound to the system. This characteristic implies that the nucleic acid had reached optimum conformation, and hence further increase in surfactant concentration did not affect its spatial arrangement. Probably, after the formation of complexes with DNA, the surfactant molecules were interacting with only themselves in more peripheral layers of formed complexes.

**Fig 5:**
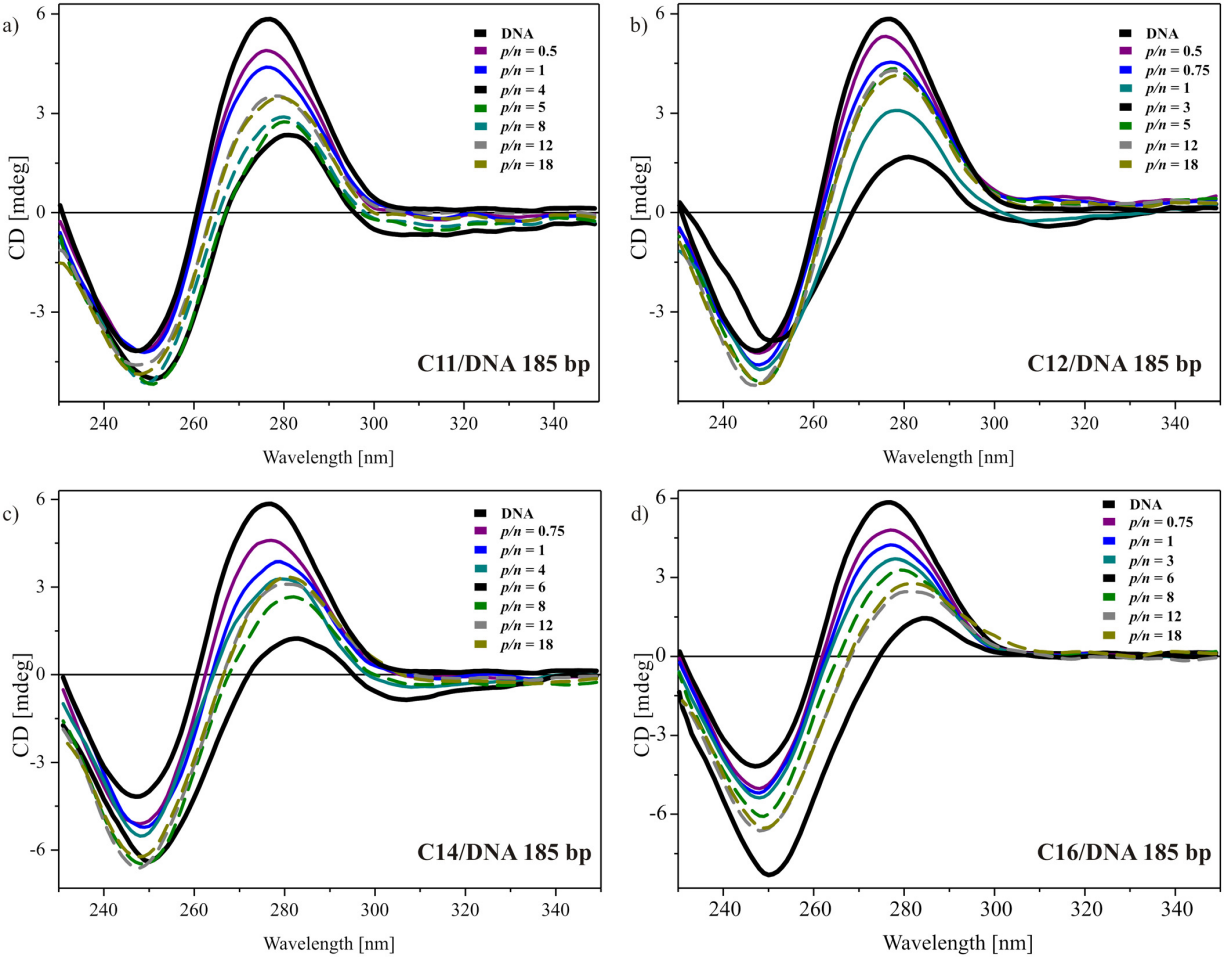
CD spectra of lipoplexes with low-molecular-weight DNA (185 base pairs). Lipoplexes based on surfactant C11 (a), C12 (b), C14 (C) and C16 (d) at different *p/n* ratios. For reasons of clarity, only selected spectra are plotted, and dotted lines were used for spectra after reaching the complexation level (those spectra are denoted based on electrophoresis results).

Fig 5 presents the CD spectra obtained for systems with low molecular salmon-sperm DNA (185 bp) and the selected, most promising surfactants C11, C12 C14 and C16. The overall tendency was similar for all mixed systems. Surfactant C11 (Fig 5a) added to DNA solution at low concentration caused a decrease in the positive band, an increase in the negative band and a redshift in the order of 2 nm. For the boundary conditions for this surfactant (*p/n* = 4), the changes were extreme and the spectrum was characterized by a maximum at 280 nm and a minimum at 250 nm, with an intersecting point at 267 nm. Additionally, the intensity of the positive ellipticity was reduced by more than 50% and the negative was increased by 20%. After reaching the complexation, the spectral changes were nullified but not to the level of the initial reference spectrum recorded for DNA solution. For *p/n* ratios higher then 10, recorded spectra were characterized by a redshift of 2 nm, with a slightly increased negative band and positive bands reaching 58% of their initial value.

A similar spectral behaviour was observed in a system based on C12 surfactant (Fig 5b), with the exception of lack of an increase in negative ellipticity in the spectrum obtained for the sample with the boundary conditions for complex formation, i.e., *p/n* = 3. For higher concentrations (*p/n* > 4), the optimum conformation of DNA was reached and the position and ellipticity of the bands remained unchanged. In case of the surfactant with the longest tails C16 (Fig 5d) the tendency was the same; however, the changes were more pronounced. For a system at *p/n* value of 6, the positive band in the CD spectrum was shifted by 6 nm, the negative band by 5 nm and the intersecting point by 15 nm, whereas the intensity of the negative ellipticity was doubled and the intensity of the positive ellipticity reached only 23% of the value obtained for the DNA solution. After complexation, the changes again progressed towards the reference spectrum, stabilizing at a point characterized by a maximum at 281 nm with half the intensity value, a minimum at 249 nm increased by 60% and the point of intersection at 268 nm. This characteristic suggests the condensation of nucleic acid, perhaps in *ψ*-form, because similar spectra have been reported for this highly condensed form of DNA [35,46].

**Fig 6:**
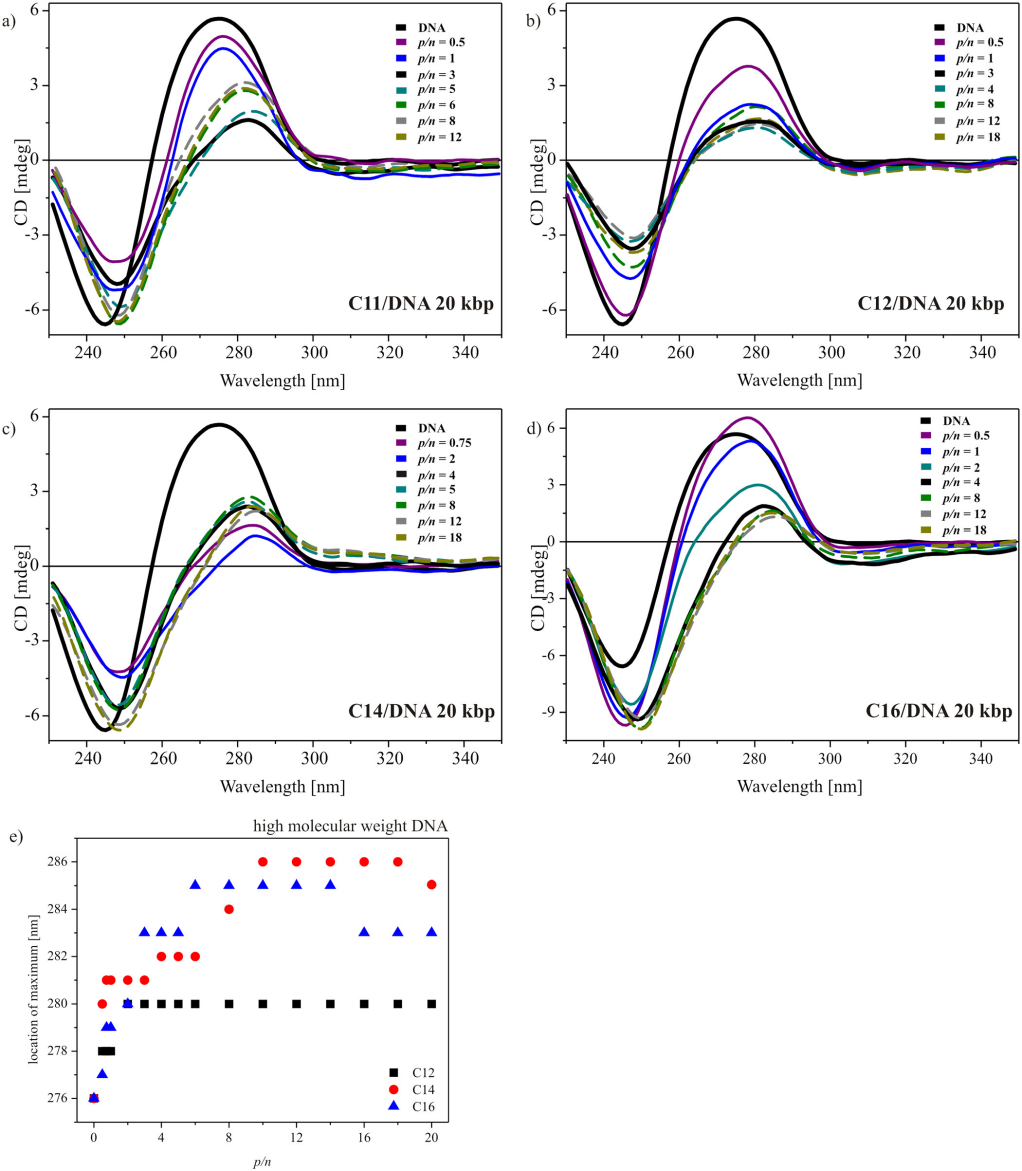
CD spectra of lipoplexes with high-molecular-weight DNA (20 kbp). Lipoplexes based on surfactant C11 (a), C12 (b), C14 (C) and C16 (d) at different *p/n* ratios. For reasons of clarity, only selected spectra are plotted, and dotted lines were used for spectra after reaching the complexation level (denoted based on electrophoresis results). The dependence of the system’s charge ratios on the location of the positive band of CD spectra for surfactants with the longest aliphatic side chains (C12, C14 and C16) (e).

Analogously, Fig 6 presents selected sets of spectra for systems with surfactants that contain long side chains (undecyloxymetyl, dodecyloxymetyl, tetradecyloxymetyl and hexadecyloxymetyl) and 20 kbp DNA. The circular dichroism spectrum of high-molecular-weight DNA solution was characterized by a maximum at 274 nm and a minimum at 245 nm, with the crossover point at 257 nm. These values indicated the native B-form of the DNA. For systems based on C11, C12 and C14 dicationic surfactants (Fig 6a, 6b and 6c, respectively), with their increasing concentration, a decrease in both bands and an overall redshift was observed. After achieving the level of complete complex formation (*p/n* = 3 or 4), the changes regressed. Most likely, the interaction between nucleic acid and gemini surfactant did not disturb the overall conformation of DNA, and the DNA’s structural integrity was retained. The observed alterations of the CD spectra originated from the modification of the DNA arrangement, such as the number of base pairs per helix and/or base angle relative to the helix axis [49] caused by changes in the hydration level and the condensation process of the nucleic acid chain [44]. A slightly different situation was visible in the case of C16 surfactant (Fig 6d), for which the negative ellipticity increased with increasing surfactant content and over *p/n* = 4, the CD spectra remained unaltered. For this system, either a conformational transition of DNA or the existence of a transition phase leading to the highly condensed phase are probable.

In general, the addition of studied dicationic surfactant molecules caused significant changes in the features of CD spectra. Both the negative and positive ellipticities’ values were affected, although the alterations of the positive band seemed to be more pronounced. Fig 6e presents the position of the maximum spectra for systems as a function of charge ratios. This band is connected with base pair stacking with chirality and is sensitive to the level of hydration of the DNA helix [44]. For the solution of DNA, the band was located at 276 nm and even a small addition of surfactant caused a shift towards a higher wavelength. This may have been the result of an ion exchange, with sodium ions adsorbed on the DNA surface. Increasing concentration of surfactants in the systems led to further redshift of this band until it reached a plateau. The position of the band steadied at 280 nm for C12 surfactant, at ~284 nm for C14 surfactant and at ~286 nm for C16 surfactant. The observed changes are explained by dehydration and/or neutralization of the phosphate group of nucleic acid and by the tightening of the base pair geometry upon the addition of surfactants and complex formation. This analysis has shown that the changes in the hydration shell of DNA correspond well with the results of electrophoretic tests because the abrupt shift of the positive band appeared at *p/n* = 5 for C14 and at *p/n* = 6 for C16. In the case of the C12 surfactant, the shift of the maximum even preceded the complete complex formation, and the plateau was reached at *p/n* = 2.

**Fig 7:**
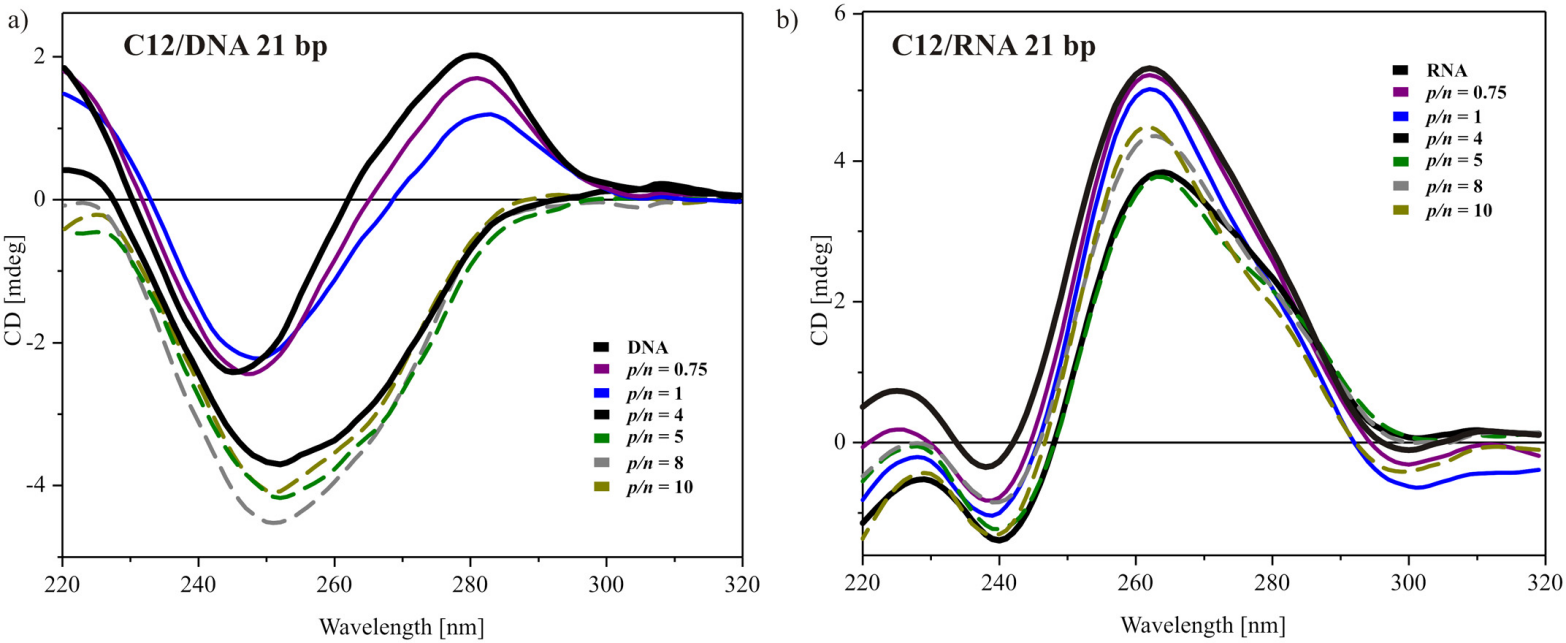
Selected CD spectra of C12 gemini surfactant with two types of oligonucleotides. Duplex DNA of 21 bp (a) and siRNA– 21 bp (b).

Circular dichroism spectra were also recorded for the smallest tested nucleic acid samples, i.e., DNA duplex and siRNA, both consisting of 21 base pairs. The results for the system based on the most efficient surfactant with dodecyloxymethyl side chains are presented in Fig 7. For DNA oligonucleotide solution, the CD spectrum (Fig 7a) was characterized by a maximum at 281 nm and a minimum at 247 nm, with an intersecting point at 262 nm. These values were slightly shifted towards higher wavelengths, and the spectrum was extended compared to the above-described spectra of low- and high-molecular-weight DNA. Nevertheless, this characteristic still points out on a typical B-form DNA in solution. The observed changes are the consequence of a specific base-pair sequence of this oligonucleotide. The addition of dicationic surfactant in quantities that did not lead to complex formation influenced the CD characteristic remotely. Essentially, only the positive ellipticity was decreased. For a system with higher surfactant content (*p/n* = 4), the positive band was flattened, whereas the intensity of the negative ellipticity was almost doubled and the band was shifted to ~252 nm. Further addition of surfactant, beyond the level of complex formation led only to a subsequent increase in the negative band’s intensity. This may be an indicative of the formation of the *ψ*-form of DNA, in which DNA helixes are oriented parallel to each other and are highly condensed [35,47,50].

The spectra recorded for the reference siRNA solution (Fig 7b) was characterised by a dominant positive peak at 260–280 nm, indicating a typical, helical A form of this nucleic acid oligomer in solution [47,49]. With an increasing surfactant concentration, a slight decrease in the intensity of this characteristic band was visible. For the system at *p/n* = 4, which is the boundary condition for complex formation, the decrease reached its maximum (~0.8 mdeg). This can be ascribed to destabilization of the base stacking of siRNA [49]. For higher surfactant content, the magnitude of the positive signal in the CD spectrum was increased and progressed to the level between the reference siRNA spectrum and the sample at *p/n* = 4. The observed changes imply that the siRNA experienced minimal structural changes upon interacting with gemini surfactant and their level indicates maintenance of the native, A-form helix of RNA in the tested range of *p/n* ratios.

**Fig 8:**
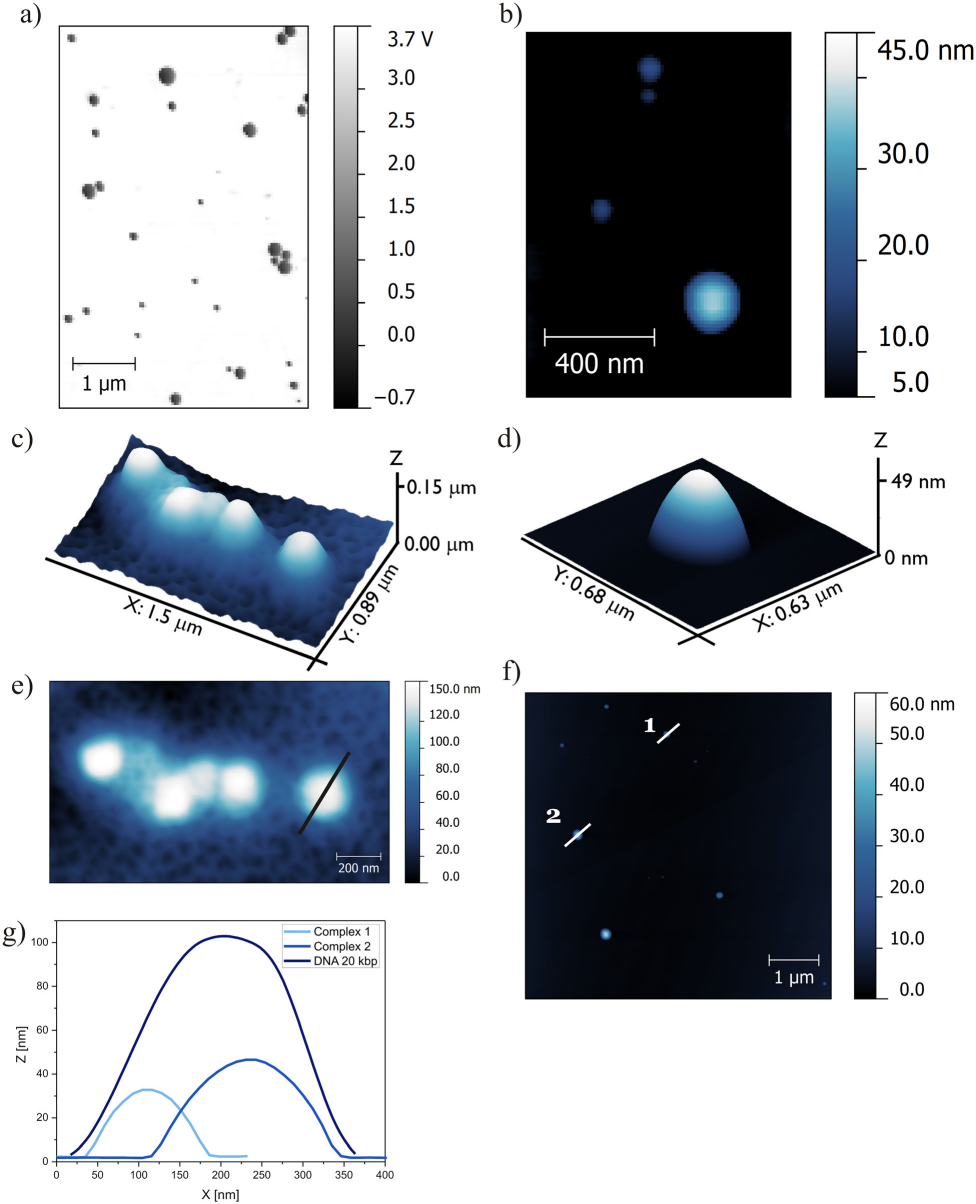
AFM topography of lipoplexes based on C12 (*p/n* = 6) and DNA (20 kbp). (a) Phase image of lipoplexes, (b) topography image of lipoplexes, (c) 3D topography of DNA reference solution (20 kbp), (d) 3D topography of complex, (e) AFM topography of DNA with cross-sections of DNA structure, (f) topography of lipoplexes with cross-sections of formed complexes, and (g) cross-sections of DNA and lipoplexes.

Atomic force microscopy has enabled visualization of the structures formed by high-molecular-weight DNA and complexes based on the most efficient gemini surfactant C12; it has also enabled estimation of the proportions of visualized aggregates. Fig 8 presents AFM images of DNA and lipoplex with the most efficient surfactant, C12 (Fig 8a, 8b and 8d), at charge ratio (*p/n* = 6), which ensures complex formation and images of the reference solution, i.e., high-molecular-weight DNA– 20 kbp (Fig 8c and 8e).

For all of the studied surfactants, because of interactions with the substrate surface and problems with the adsorption of prepared solutions, it was not possible to image the reference surfactants solutions. This problem was probably caused by the amphiphilic character and very strong surface activity of gemini surfactants, and therefore, this type of sample should be visualized by measurements in buffer solution. The reference sample/solution of high-molecular-weight salmon-sperm DNA adsorbed on the mica surface (without divalent cations) formed coil-like structures with a spherical shape. To visualize the lipoplexes with C12 surfactant, the system with a charge ratio *p/n* of 6 was chosen based on the gel electrophoresis results indicating that stable complexes were formed at this value. For this system, observed complexes had a similar spherical shape with a broad size distribution. Several large aggregates were visible and were characterized by their proportions in the z-dimension on the order of 200 nm and their size in the xy dimensions in the order of 500 nm. It is probable that these large aggregates were formed by one or several additional layers of surfactant being built on the complex to avoid both unfavourable hydrophobic interactions and the flattering effect of sample-mica interactions. Medium- and small-size aggregates were selected for further analysis.

**Fig 9:**
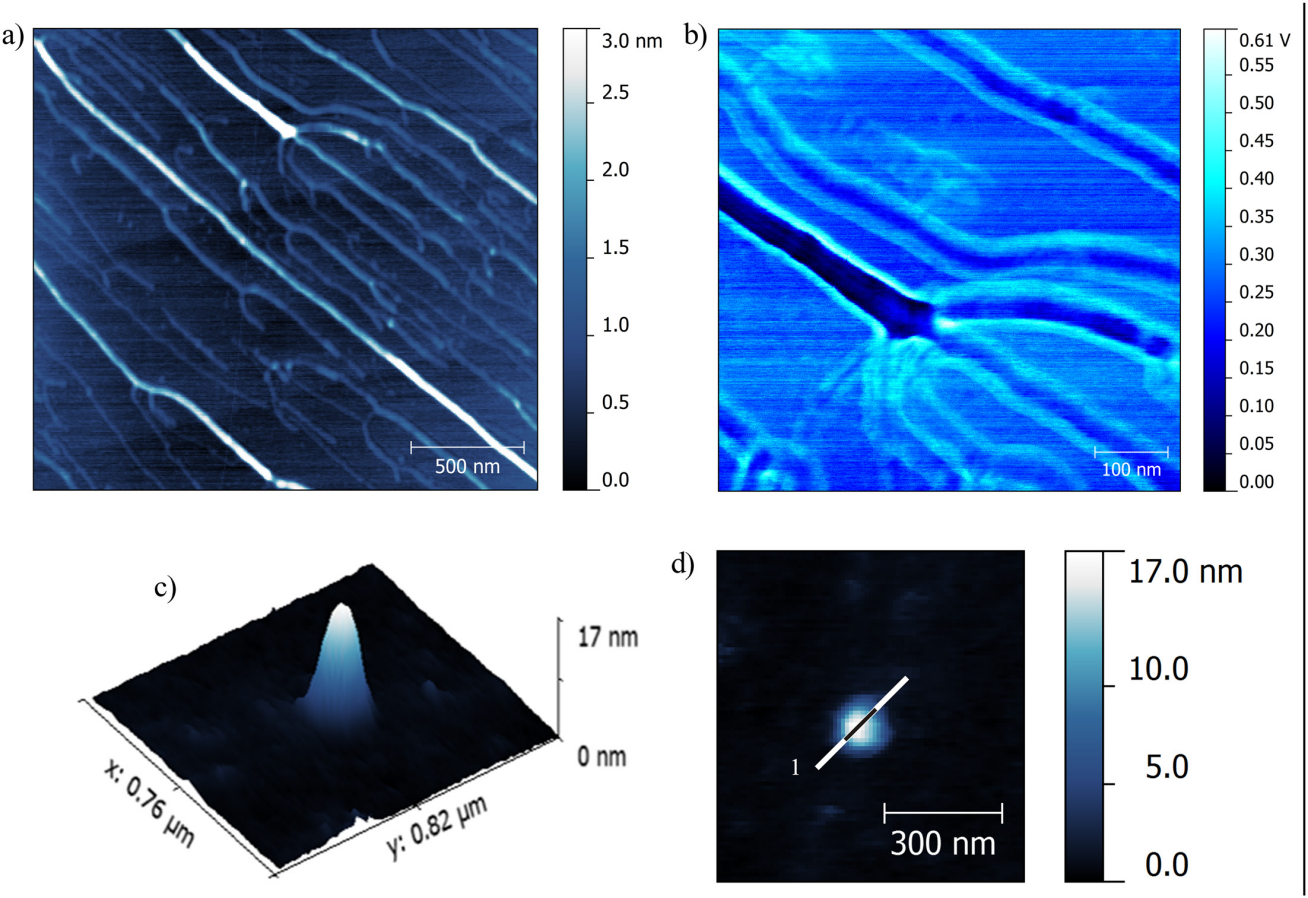
AFM results of lipoplexes based on C6 surfactant. AFM images of system based on C6 surfactant at a *p/n* ratio of 0.75 and high -MW DNA for which complex formation was not detected. On the left (a), AFM topography of observed extended structures and phase image on the right (b). The dark fields probably consist of DNA molecules, which are surrounded by surfactant molecules/bright fields. Below AFM images of lipoplex based on surfactant C12 and DNA oligonucleotide at *p/n* = 5. 3D view (c) and topography with marked cross-section (d).

On the basis of topographies shown in Fig 8e and 8f with marked cross-section spots and the cross-sections profiles themselves (Fig 8g), the average size of high-MW DNA and lipoplexes formed with C12 were estimated. The height of the DNA-entangled aggregate was an average of 100 nm, and the size of the lipoplexes was in the range of 30–50 nm. Based on these values, we can conclude that the process of complexation is accompanied by condensation of DNA and therefore, lipoplexes have smaller dimensions than the initial DNA coils. It has been reported previously [38] that the same high-molecular-weight DNA can adsorb on the mica in the form of long strands of DNA with lengths that exceed 500 nm. These findings were in good agreement with those from other studies [51]. For that reason, the imaging was conducted on the system containing DNA and gemini surfactant at low concentration, for which no complexes were formed. AFM images of samples consisting of high-MW DNA and gemini surfactant C6 at a *p/n* ratio of 0.75 are presented in Fig 9a and 9b.

AFM topography revealed the extended fibre-like structures in the sample that proved ineffective at forming complexes with DNA. There were two main types of structures with different sizes. The thinnest fibre-like structures had a height of approximately 1–2 nm and a width of approximately 30 nm, whereas the thicker fibres had a height of 10 nm and a width of approximately 100 nm. These results imply that addition of the studied gemini surfactant caused uncoiling of the DNA strands, although the interactions were insufficient to form a stable complex with nucleic acid. As seen in the phase image (Fig 9b), the fibre-like structure is not homogeneous, i.e., the material forming the periphery has different mechanical properties than those of the inner material. The phase imaging is explained in terms of the energy dissipation during the contact between the tip and the sample. Therefore, it depends on factors such as viscoelasticity or adhesion and enables visualization of the distribution of different phases within the sample. Therefore, we can conclude that the inner material consists of DNA molecules visible on the phase image as dark fields with a bright layer of surfactants stabilizing the elongated structures on the edges. Similar phase images have been reported for lipoplexes with siRNA, for which cationic complexes were visualized, surrounded by bright coverage formed by PEG molecules [52]. This would confirm the hypothesis that surfactants lead to unfolding of the coil-like structures observed in the reference DNA solution.

The AFM technique has also been applied to visualize the lipoplexes containing DNA duplex consisting of 21 base pairs. Nevertheless, obtaining an AFM image for this DNA’s reference solution was not possible, probably because of the small size of this nucleic acid and the limitation of the resolution of the applied measuring setup. In the case of lipoplexes based on surfactant C12, the flattened, spherical structures were mostly small vesicles of ∼100–150 nm (dimension in xy plane) with heights of approximately 10–20 nm. The singular complex of C12 and DNA duplex with a charge ratio *p/n* of 5 is presented in the Fig 9 as 3D view (c) and topography with marked cross-section (d).

**Fig 10:**
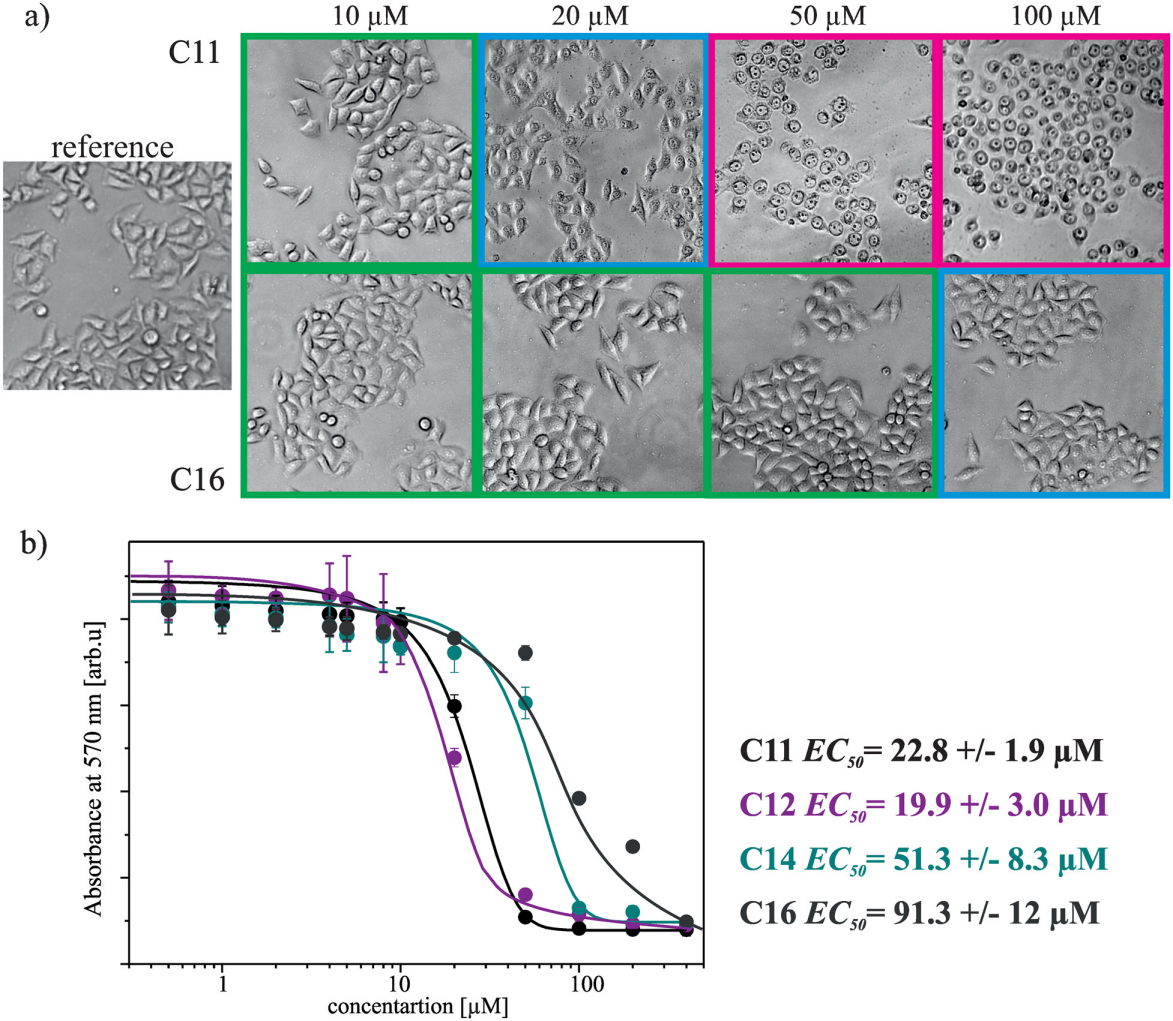
Results of cytotoxicity tests. Selected images of HeLa cells without any treatment (reference sample) and after exposure to solutions of gemini surfactants (C11 and C16), at different concentrations. Below, the dependence of absorbance measured at 570 nm on surfactants concentration, after MTT assay with fitted Boltzmann models.

Additionally to structural studies of lipoplexes we also evaluated the cytotoxicity of studied gemini surfactants. For the quantitative analysis of the cytotoxicity by the use of MTT tests were selected four surfactants: C11, C12, C14 and C16 and dose-response curves for each surfactant are presented in Fig 10. As a reference to MTT tests in Fig 10 are also presented the morphology changes of HeLa cells in different concentrations of studied surfactants (denoted as surfactant concentration used in the cell culture).

The observed morphological changes, induced by surfactant present in the cell culture medium, are dependent on the length of surfactant hydrophobic chain. It is worth be noted that the studied surfactants possessing short hydrophobic side chains (e.g. C11) induce significant morphological changes of cells already in a concentration of 20 μM, however surfactant possessing the longest hydrophobic chain (C16) induces similar changes at the concentration of 100 μM. An identical dependence between the geometry and the toxicity of tested surfactants illustrate the results of MTT assays, wherein the observed EC_50_ value for surfactant C12 is 19.9 μM, however the surfactant C16 is characterised by EC_50_ of 91.3 μM. Such cytotoxicity values promote the studied surfactants for further extensive transfection tests.

## Conclusions

The studied gemini surfactants are promising components of new nucleic acid delivery systems for use in gene therapy. To form complexes with nucleic acids, side chains are required to be a particular length. In the case of the tested series of surfactants, the hydrophobic chains need to be longer than 9 carbon atoms (nonyloxymethyl side-chain group), with an optimum value of 12 atoms (dodecyloxymethyl side-chain group), at which the effectiveness of the surfactant that binds various nucleic acids has been the highest. The tested surfactants formed stable complexes with low- and high-molecular-weight salmon-sperm DNA and 21 bp DNA and RNA at specific concentrations, i.e., *p/n* values. The size of the nucleic acid used also affected the process of complexation. The bigger the nucleic acid molecules, the less amount of amphiphiles were needed to form stable complexes.

Structural changes in the lipoplexes’ formations were also observed, with CD spectra showing that as the concentration of gemini surfactants increases, the DNA becomes condensed and most likely maintains its native B-form. However, the formation of a highly condensed *ψ* phase of DNA cannot be completely excluded. Moreover, for high-MW DNA lipoplex, the formation of the *ψ* phase is more likely. AFM imaging confirmed the findings of the CD experiments, and the condensation of DNA molecules and spherically shaped complexes were observed. Surfactants that were unable to form stable complexes were proven to interact with DNA by covering elongated DNA structures.

The presented study broadens the understanding of lipoplex formation and the interaction of gemini surfactants with various types of nucleic acids, which hopefully will lead to improvement of the screening of the amphiphilic molecules and prediction of their potential applicability in gene therapy.

## References

[1] Janssens ACJW, van Duijn CM. Genome-based prediction of common diseases: advances and prospects Hum. Mol. Genet. 2008; 17 (R2): R166–R173. 10.1093/hmg/ddn250 18852206

[2] Verma M. Personalized Medicine and Cancer. J. Pers. Med. 2012; 2: 1–14. 10.3390/jpm2010001 25562699 PMC4251363

[3] Emery DW. Gene therapy for genetic diseases: On the horizon. Clin Appl Immunol Rev. 2004;4: 411–422.

[4] Ibraheem D, Elaissari A, Fessi H. Gene therapy and DNA delivery systems. Int J Pharm. 2014;459: 70–83. 10.1016/j.ijpharm.2013.11.041 24286924

[5] Maguire AM, Simonelli F, Pierce EA, Pugh EN, Mingozzi F, Bennicelli J, et al Safety and Efficacy of Gene Transfer for Leber’s Congenital Amaurosis. N Engl J Med. 2008;358: 2240–2248. 10.1056/NEJMoa0802315 18441370 PMC2829748

[6] LeWitt PA, Rezai AR, Leehey MA., Ojemann SG, Flaherty AW, Eskandar EN, et al AAV2-GAD gene therapy for advanced Parkinson’s disease: a double-blind, sham-surgery controlled, randomised trial. Lancet Neurol. 2011;10: 309–319. 10.1016/S1474-4422(11)70039-4 21419704

[7] Chen G-X, Zhang S, He X, Liu S, Ma C, Zou X-P. Clinical utility of recombinant adenoviral human p53 gene therapy: current perspectives. Onco Targets Ther. 2014; 7: 1901–1909. 10.2147/OTT.S50483 25364261 PMC4211860

[8] Behlke M. Progress towards in Vivo Use of siRNAs. Mol Ther. 2006;13: 644–670. 16481219 10.1016/j.ymthe.2006.01.001 PMC7106286

[9] Gandhi NS, Tekade RK, Chougule MB. Nanocarrier mediated delivery of siRNA/miRNA in combination with chemotherapeutic agents for cancer therapy: Current progress and advances. J Controlled Release. 2014;194: 238–256. 10.1016/j.jconrel.2014.09.001 PMC4254052 25204288

[10] Li S, Huang L. Nonviral gene therapy: promises and challenges. Gene Ther. 2000;7: 31–34. 10680013 10.1038/sj.gt.3301110

[11] Giacca M, Zacchigna S. Virus-mediated gene delivery for human gene therapy. J Controlled Release. 2012;161: 377–388. 10.1016/j.jconrel.2012.04.008 22516095

[12] Donkuru M, Badea I, Wettig S, Verrall R, Elsabahy M, Foldvari M. Advancing nonviral gene delivery: lipid- and surfactant-based nanoparticle design strategies. Nanomed. 2010;5: 1103–1127. 10.2217/nnm.10.80 20874024

[13] In M, Zana R. Phase Behavior of Gemini Surfactants. J Dispers Sci Technol. 2007;28: 143–154.

[14] Zana R. Dimeric and oligomeric surfactants. Behavior at interfaces and in aqueous solution: a review. Adv Colloid Interface Sci. 2002;97: 205–253. 12027021 10.1016/s0001-8686(01)00069-0

[15] Ilies MA, Seitz WA, Johnson BH, Ezell EL, Miller AL, Thompson EB, et al Lipophilic Pyrylium Salts in the Synthesis of Efficient Pyridinium-Based Cationic Lipids, Gemini Surfactants, and Lipophilic Oligomers for Gene Delivery. J Med Chem. 2006;49: 3872–3887. 16789743 10.1021/jm0601755

[16] Karlsson L, van Eijk MCP, Söderman O. Compaction of DNA by Gemini Surfactants: Effects of Surfactant Architecture. J Colloid Interface Sci. 2002;252: 290–296. 16290792 10.1006/jcis.2002.8477

[17] Muñoz-Úbeda M, Misra SK, Barrán-Berdón AL, Datta S, Aicart-Ramos C, Castro-Hartmann P, et al How Does the Spacer Length of Cationic Gemini Lipids Influence the Lipoplex Formation with Plasmid DNA? Physicochemical and Biochemical Characterizations and their Relevance in Gene Therapy. Biomacromolecules. 2012; 121116090025006. 10.1021/bm301066w 23130552

[18] Kirby AJ, Camilleri P, Engberts JBFN, Feiters MC, Nolte RJM, Söderman O, et al Gemini Surfactants: New Synthetic Vectors for Gene Transfection. Angew Chem Int Ed. 2003;42: 1448–1457. 10.1002/anie.200201597 12698476

[19] Zana R. Alkanediyl-α,ω-bis(dimethylalkylammonium bromide) Surfactants. J Colloid Interface Sci. 2002;246: 182–190. 16290399 10.1006/jcis.2001.7921

[20] Zana R, Benrraou M, Rueff R. Alkanediyl-.alpha.,.omega.-bis(dimethylalkylammonium bromide) surfactants. 1. Effect of the spacer chain length on the critical micelle concentration and micelle ionization degree. Langmuir. 1991;7: 1072–1075.

[21] Kamboj R, Singh S, Bhadani A, Kataria H, Kaur G. Gemini Imidazolium Surfactants: Synthesis and Their Biophysiochemical Study. Langmuir. 2012;28: 11969–11978. 10.1021/la300920p 22845861

[22] Ristori S, Ciani L, Candiani G, Battistini C, Frati A, Grillo I, et al Complexing a small interfering RNA with divalent cationic surfactants. Soft Matter. 2012;8: 749.

[23] Falsini S, Ristori S, Ciani L, Di Cola E, Supuran CT, Arcangeli A, et al Time resolved SAXS to study the complexation of siRNA with cationic micelles of divalent surfactants. Soft Matter. 2013;10: 2226–2233. 10.1039/c3sm52429a 24651873

[24] Badea I, Verrall R, Baca-Estrada M, Tikoo S, Rosenberg A, Kumar P, et al In vivo cutaneous interferon-γ gene delivery using novel dicationic (gemini) surfactant-plasmid complexes. J Gene Med. 2005;7: 1200–1214. 15895387 10.1002/jgm.763

[25] Rosenzweig HS, Rakhmanova VA, MacDonald RC. Diquaternary Ammonium Compounds as Transfection Agents. Bioconjug Chem. 2001;12: 258–263. 11312687 10.1021/bc000099z

[26] Wang C, Li X, Wettig SD, Badea I, Foldvari M, Verrall RE. Investigation of complexes formed by interaction of cationic gemini surfactants with deoxyribonucleic acid. Phys Chem Chem Phys. 2007;9: 1616 17429555 10.1039/b618579g

[27] Kumar K, Barrán-Berdón AL, Datta S, Muñoz-Úbeda M, Aicart-Ramos C, Kondaiah P, et al A delocalizable cationic headgroup together with an oligo-oxyethylene spacer in gemini cationic lipids improves their biological activity as vectors of plasmid DNA. J Mater Chem B. 2015;3: 1495–1506. 10.1039/c4tb01948b 32262422

[28] Pietralik Z, Taube M, Skrzypczak A, Kozak M. SAXS Study of Influence of Gemini Surfactant, 1, 1’-(1, 4-butanediyl) bis 3-cyclododecyloxymethylimidazolium dichloride, on the Fully Hydrated DMPC. Acta Phys Pol A. 2010;117: 311–314.

[29] Nečas D, Klapetek P. Gwyddion: an open-source software for SPM data analysis. Cent Eur J Phys. 2012;10: 181–188.

[30] Mosmann T. Rapid colorimetric assay for cellular growth and survival: Application to proliferation and cytotoxicity assays. J Immunol Methods. 1983;65: 55–63. 6606682 10.1016/0022-1759(83)90303-4

[31] Optimized Protocol for HeLa Cells [ATCC^®^]. Lonza Cologne AG, Cologne, Germany.

[32] Khatri N, Misra A. Development of siRNA lipoplexes for intracellular delivery in lung cancer cells. J Pharm Bioallied Sci. 2012;4(Suppl 1):S1–3. 10.4103/0975-7406.94115 23066176 PMC3467833

[33] Muñoz-Úbeda M, Misra SK, Barrán-Berdón AL, Datta S, Aicart-Ramos C, Castro-Hartmann P, et al How Does the Spacer Length of Cationic Gemini Lipids Influence the Lipoplex Formation with Plasmid DNA? Physicochemical and Biochemical Characterizations and their Relevance in Gene Therapy. Biomacromolecules. 2012;13:3926–3937. 10.1021/bm301066w 23130552

[34] Sharma VD, Ilies MA. Heterocyclic Cationic Gemini Surfactants: A Comparative Overview of Their Synthesis, Self-assembling, Physicochemical, and Biological Properties: Heterocyclic Cationic Gemini Surfactants. Med Res Rev. 2014;34: 1–44. 10.1002/med.21272 22907528

[35] Bombelli C, Borocci S, Diociaiuti M, Faggioli F, Galantini L, Luciani P, et al Role of the spacer of cationic gemini amphiphiles in the condensation of DNA. Langmuir. 2005;21: 10271–10274. 16262274 10.1021/la051324+

[36] Luciani P, Bombelli C, Colone M, Giansanti L, Ryhänen SJ, Säily VMJ, et al Influence of the Spacer of Cationic Gemini Amphiphiles on the Hydration of Lipoplexes. Biomacromolecules. 2007;8: 1999–2003. 17518442 10.1021/bm070202o

[37] Barrán-Berdón AL, Misra SK, Datta S, Muñoz-Úbeda M, Kondaiah P, Junquera E, et al Cationic gemini lipids containing polyoxyethylene spacers as improved transfecting agents of plasmid DNA in cancer cells. J Mater Chem B. 2014;2: 4640–4652. 10.1039/c4tb00389f 32262276

[38] Pietralik Z, Kumita JR, Dobson CM, Kozak M. The influence of novel gemini surfactants containing cycloalkyl side-chains on the structural phases of DNA in solution. Colloids Surf B Biointerfaces. 2015;131: 83–92. 10.1016/j.colsurfb.2015.04.042 25969417

[39] Muñoz-Úbeda M, Misra SK, Barrán-Berdón AL, Aicart-Ramos C, Sierra MB, Biswas J, et al Why Is Less Cationic Lipid Required To Prepare Lipoplexes from Plasmid DNA than Linear DNA in Gene Therapy? J Am Chem Soc. 2011;133: 18014–18017. 10.1021/ja204693f 21985329

[40] Dan N, Danino D. Structure and kinetics of lipid–nucleic acid complexes. Adv Colloid Interface Sci. 2014;205: 230–239. 10.1016/j.cis.2014.01.013 24529969

[41] Wettig SD, Badea I, Donkuru M, Verrall RE, Foldvari M. Structural and transfection properties of amine-substituted gemini surfactant-based nanoparticles. J Gene Med. 2007;9: 649–658. 17654656 10.1002/jgm.1060

[42] Karlsson L, van Eijk MCP, Söderman O. Compaction of DNA by Gemini Surfactants: Effects of Surfactant Architecture. J Colloid Interface Sci. 2002;252: 290–296. 16290792 10.1006/jcis.2002.8477

[43] Bajaj A, Kondaiah P, Bhattacharya S. Synthesis and Gene Transfer Activities of Novel Serum Compatible Cholesterol-Based Gemini Lipids Possessing Oxyethylene-Type Spacers. Bioconjug Chem. 2007;18: 1537–1546. 17661441 10.1021/bc070010q

[44] Zhou T, Xu G, Ao M, Yang Y, Wang C. DNA compaction to multi-molecular DNA condensation induced by cationic imidazolium gemini surfactants. Colloids Surf Physicochem Eng Asp. 2012;414: 33–40.

[45] Pietralik Z, Krzysztoń R, Kida W, Andrzejewska W, Kozak M. Structure and Conformational Dynamics of DMPC/Dicationic Surfactant and DMPC/Dicationic Surfactant/DNA Systems. Int J Mol Sci. 2013;14: 7642–7659. 10.3390/ijms14047642 23571492 PMC3645708

[46] Zhao X, Shang Y, Liu H, Hu Y, Jiang J. Interaction of DNA with Cationic Gemini Surfactant Trimethylene-1, 3-bis(dodecyldimethyl-ammonium bromide) and Anionic Surfactant SDS Mixed System. Chin J Chem Eng. 2008;16: 923–928.

[47] Kypr J, Kejnovska I, Renciuk D, Vorlickova M. Circular dichroism and conformational polymorphism of DNA. Nucleic Acids Res. 2009;37: 1713–1725. 10.1093/nar/gkp026 19190094 PMC2665218

[48] Braun CS, Kueltzo LA, Middaugh CR. Ultraviolet Absorption and Circular Dichroism Spectroscopy of Nonviral Gene Delivery Complexes Nonviral Vectors for Gene Therapy. New Jersey: Humana Press; 2001 pp. 253–284. 10.1385/1-59259-139-6:253 21318759

[49] O’Mahony AM, Cronin MF, Mcmahon A, Evans JC, Daly K, Darcy R, et al Biophysical and Structural Characterisation of Nucleic Acid Complexes with Modified Cyclodextrins Using Circular Dichroism. J Pharm Sci. 2014;103: 1346–1355. 10.1002/jps.23922 24604260

[50] Bello-Roufai M, Lambert O, Pitard B. Relationships between the physicochemical properties of an amphiphilic triblock copolymers/DNA complexes and their intramuscular transfection efficiency. Nucleic Acids Res. 2007;35: 728–739. 17182627 10.1093/nar/gkl860 PMC1807968

[51] Adamcik J, Klinov DV, Witz G, Sekatskii SK, Dietler G. Observation of single-stranded DNA on mica and highly oriented pyrolytic graphite by atomic force microscopy. FEBS Lett. 2006;580: 5671–5675. 17007844 10.1016/j.febslet.2006.09.017

[52] Belletti D, Tonelli M, Forni F, Tosi G, Vandelli MA, Ruozi B. AFM and TEM characterization of siRNAs lipoplexes: A combinatory tools to predict the efficacy of complexation. Colloids Surf Physicochem Eng Asp. 2013;436: 459–466.

